# Granular cell tumor of the thyroid: Clinical and pathological characteristics of a rare case in a 14-year-old girl

**DOI:** 10.3892/ol.2014.2775

**Published:** 2014-12-08

**Authors:** ZHEN-HONG DU, HONG-YAN QIU, TAO WEI, JING-QIANG ZHU

**Affiliations:** 1 Department of Gastrointestinal and Thoracic Surgery, 363 Hospital, West China Hospital of Sichuan University, Chengdu, Sichuan 610041, P.R. China; 2 Department of Infectious Disease, 416 Hospital, West China Hospital of Sichuan University, Chengdu, Sichuan 610041, P.R. China; 3 Department of Thyroid and Breast Surgery, West China Hospital of Sichuan University, Chengdu, Sichuan 610041, P.R. China

**Keywords:** granular cell tumor, thyroid, atypical, immunohistochemical staining

## Abstract

Granular cell tumors (GCTs) are soft tissue neoplasms that originate in the nervous system, which may arise anywhere in the body. However, GCTs are extremely uncommon in thyroid tumors, with a favorable prognosis. The diagnosis of GCTs is dependent on pathological and immunohistochemical analysis and at present, surgical resection is considered the only suitable treatment. Regular follow-up after surgery is an important way to monitor treatment outcome and recurrence. The present study describes a new pathological type of thyroid GCTs diagnosed by pathology and immunohistochemistry. A 14-year-old female was referred to the West China Hospital of Sichuan University (Chengdu, China), for thyroid incidentaloma. Laboratory examinations were within the normal range. Thyroid sonography demonstrated a solid hypoechoic mass in the right lobe of the thyroid. Fine needle aspiration cytology showed a suspicious malignant tumor and subsequently a total thyroidectomy was performed. Analysis of frozen sections, from obtained samples, did not facilitate a definite diagnosis. Finally, a thyroid benign granular tumor with atypical changes was diagnosed by postoperative pathology and immunohistochemistry. A 14-month post-operative follow-up showed that the patient experienced a stable recovery and had no signs of recurrence or metastasis. The case emphasizes that the diagnosis of thyroid granular cell tumors is predominantly based on postoperative morphology and immunophenotype. The clinical routine for the differential diagnosis may be due to: (i) neoplasms displaying a granular appearance mimicking granular cell tumors, or (ii) differential diagnosis in the pathological category of granular cell tumors. Further accumulation of such rare cases may be of clinical significance in aiding the diagnosis and treatment of GCTs.

## Introduction

Granular cell tumors (GCTs) are rare soft tissue neoplasms that were first reported by Abrikossoff in 1926 (1). GCTs have been frequently identified in various organs including the breast, urinary bladder, testis, ovary, esophagus, heart, head and neck (2,3). The incidence rate of thyroid GCTs is extremely low. Only 11 cases have been reported in the English language literature (3–13). The rarity of thyroid GCTs and characteristics mimicking malignancy results in a diagnostic challenge for surgeons on clinical examination. Thyroid GCTs are therefore unlikely to be diagnosed correctly on initial examination unless pathological and immunohistochemical results are available (5,13). Numerous lines of evidence are required to contribute to an accurate diagnosis. Even though the number of thyroid GCTs is increasing, our understanding of these tumors requires further research. An increase in the number of cases of thyroid GCTs is expected to be publically reported. The present case study describes a novel case of thyroid GCT, with a description of the primary aspects of its clinical and pathological characteristics. Written informed consent was obtained from the patient’s family.

## Case report

### Patient history

A 14-year-old Chinese female was referred to the West China Hospital of Sichuan University (Chengdu, China), for evaluation of a thyroid incidentaloma. The patient presented with a 3-month history of a neck lump without thyrotoxicosis or symptoms of hypothyroidism. The mass (~2.5×2.0 cm) was located in the right thyroid lobe without cervical lymphadenectasis.

### Patient examination

Laboratory examinations, including thyroid function tests, carcinoembryonic antigen, calcitonin and serum calcium levels were in the normal range. A thyroid ultrasound disclosed that the mass consisted of numerous thyroid nodules, and the biggest nodule showed an irregular shape, infiltrative margins, intranodular avascularity and a shape taller than width. The mass was subsequently classified as grade 5 according to the Thyroid Imaging Reporting and Data System (14). Furthermore, fine needle aspiration cytology of the thyroid nodule indicated a high likelihood of malignancy. The patient had no history of family thyroid disease or external irradiation.

### Diagnosis and treatment

**Figure 1:**
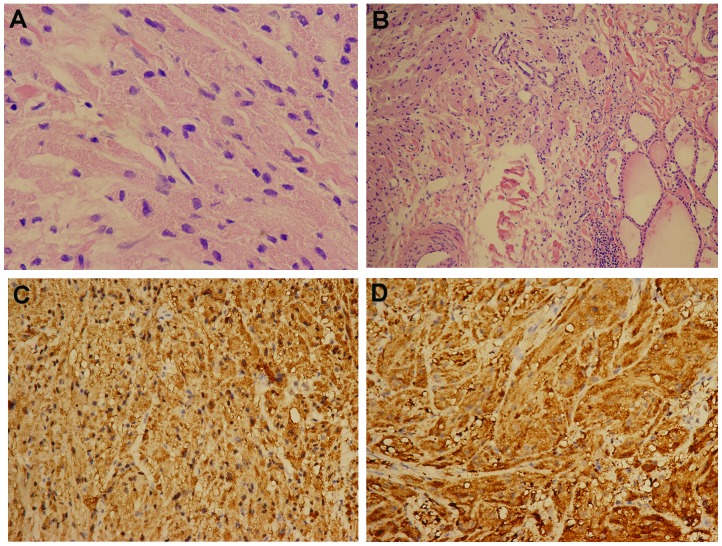
Histological features of a benign granular cell tumor with atypical changes. (A) Nests of epithelioid cells with an oval or spindle deep-dyed large, hyperchromatic nucleolus and an abundant granular eosinophilic cytoplasm are observed (staining, hematoxylin and eosin; magnification, ×350). (B) The granular cell tumor was interdigitated with adjacent thyroid follicles (staining, hematoxylin and eosin; magnification, ×200). (C) The granular cell tumor was positive for S-100 protein on immunohistochemical staining (staining, hematoxylin and eosin; magnification, ×350). (D) The granular cell tumor was positive for neuron-specific enolase on immunohistochemical staining (staining, hematoxylin and eosin; magnification, ×350).

Taken together, these features gave rise to a diagnosis for malignancy and a thyroidectomy was performed. Intraoperatively, the frozen section revealed a tumor that was derived from epithelial or mesenchymal tissue. Macroscopically, the tumor had progressively invaded the surrounding tissues and resembled a follicular neoplasm. The tumor was histopathologically characterized by nests of epithelioid cells with an oval or spindle deep-dyed large, hyperchromatic nucleolus and an abundant granular eosinophilic cytoplasm. A few of the cells showed heteromorphism, and there was no mitotic activity (Fig. 1A). The nests were separated by a septate fiber, as well as interdigitated with surrounding thyroid follicles (Fig. 1B) and the adjacent ipsilateral cricothyroid muscle. Immunohistochemical analysis indicated that the tumor cells originated from Schwann cells due to positive staining for S-100 protein (Fig. 1C), neuron-specific enolase (Fig. 1D) and CD68. The tumor cells were negative for thyroid transcription factor-1 and thyroglobulin.

### Postoperative outcomes

Postoperatively, the clinical course of the patient was uneventful. Medication with levothyroxine at a daily dose of 62.5 μg was only administered in order to maintain the serum thyrotropin at a level <0.5 mU/l. Chemotherapy or radioiodine treatment was not performed. The patient had no recurrence and remained healthy during the 14-month post-operative follow-up visit.

## Discussion

GCTs are rare soft tissue neoplasms, which may occur at any age and at most anatomical locations (2). GCTs of the thyroid, however, are extremely rare. Due to the rarity of the lesion, preoperative and intraoperative diagnoses of thyroid GCTs remain challenging (3–5,8–10,15–17). Clinically, the majority of patients are asymptomatic (4–13). One case has been reported of nonspecific symptoms, such as weakness, anxiety and palpitation (3). Laboratory examinations, including thyroid function tests, carcinoembryonic antigen, calcitonin and serum calcium levels, are useful for differential diagnosis but are nonspecific. The gold standard for diagnosis of GCTs depends on the results obtained from pathological and immunohistochemical analysis. The criteria to distinguish between pathological types of GCT are necrosis, spindling, vesicular nuclei with prominent nucleoli, increased mitotic activity (>2/10 high-power fields), high nuclear/cytoplasmic ratio and pleomorphism (17). Tumors with three or more of these criteria are considered to have malignant behavior. Those with one or two features are termed as ‘atypical granular cell tumors’. Cases that meet none of these criteria, or have only a focal pleomorphism, are classified as benign. The clinical routine of differential diagnosis of thyroid GCTs occurs predominantly from two aspects. Firstly, a number of benign/malignant tumors display a granular appearance, mimicking GCTs, such as Hurthle cell papillary thyroid carcinoma, follicular Hurthle cell tumor of the thyroid, as well as medullary thyroid cancer. These tumors need to be excluded from the diagnosis. Secondly, the distinction in pathological category of granular cell tumors is essential. GCTs were initially considered to be myogenic in origin, but high positive rates for S-100 protein and neuron-specific enolase detected by immunohistochemistry suggests that a Schwann cell derivation is more likely (15). At present, surgical excision is the only treatment option (18), and to date, radiation or chemotherapy as not been proposed. Most thyroid GCTs are benign, and patients have a favorable prognosis (9). Malignant GCTs are relatively uncommon, constituting 1–2% of all granular cell tumors (16), and there is little information on the response of malignant GCTs to therapeutic modalities, other than surgical intervention. Local recurrence has been reported after incomplete excision as early as 3 months after surgical removal of the tumor (13). Follow-up examinations are vital to observe the curative effects.

In the present case, the clinical manifestations and laboratory examinations were not unusual. The ultrasound raised suspicion of the possibility of a thyroid carcinoma, and fine needle aspiration cytology of the thyroid nodule indicated a high likelihood of malignancy. A surgical intervention was performed since a tumor was suspected on fine needle aspiration biopsy. Sections analyzed from the intraoperative frozen tumor specimen failed to facilitate a diagnosis. A thyroid GCT was finally diagnosed by postoperative pathology and immunohistochemistry. According to the six histological criteria for assessing the character of thyroid GCTs (3), the presenting case was classified as a benign GCT with atypical changes, since none of the criteria were met, other than focal pleomorphism and occasional spindle cell. The specimen did not meet the standard of atypical GCTs. Hematoxylin and eosin-stained sections showed a partially circumscribed neoplasm, consisting of oval to spindle cells, a hyperchromatic nucleus and an abundant granular eosinophilic cytoplasm, as well as a few of the cells displaying heteromorphism (a large nucleus and dense cytoplasm) without mitotic activity. Immunohistochemical analysis showed positive expression of S-100, neuron-specific enolase and CD68, but was negative for thyroid transcription factor-1 and thyroglobulin. The tumor was surgically removed, and no radiotherapy or chemotherapy was administered except for a routine medication following the surgery (oral levothyroxine at a daily dose of 62.5 μg). Post-operative follow-up at 14 months showed that the patient had experienced a stable recovery with no signs of recurrence or metastasis.

In conclusion, thyroid GCT is an extremely rare and highly aggressive tumor with a favorable prognosis. Clinically, the diagnosis of thyroid GCTs is based on pathology and immunohistochemistry and surgical resection is currently considered as the only treatment. In the presented case, routine postoperative medical management proved efficient, and a long-term follow-up was performed in order to confirm the outcome of these treatments. Further accumulation of the clinical and histopathological features of such rare cases may be of clinical significance in the diagnosis and treatment of thyroid GCTs.
